# Inhibitory effects of vaginal *Lactobacilli* on C*andida albicans* growth, hyphal formation, biofilm development, and epithelial cell adhesion

**DOI:** 10.3389/fcimb.2023.1113401

**Published:** 2023-05-02

**Authors:** Tomonori Takano, Hayami Kudo, Shuhei Eguchi, Asami Matsumoto, Kentaro Oka, Yukitaka Yamasaki, Motomichi Takahashi, Takuro Koshikawa, Hiromu Takemura, Yuka Yamagishi, Hiroshige Mikamo, Hiroyuki Kunishima

**Affiliations:** ^1^Department of Infectious Diseases, St. Marianna University School of Medicine, Kawasaki-shi, Kanagawa, Japan; ^2^Research Department, R&D Division, Miyarisan Pharmaceutical Co., Ltd., Saitama-shi, Saitama, Japan; ^3^Department of Microbiology, St. Marianna University School of Medicine, Kawasaki-shi, Japan; ^4^Department of Clinical Infectious Diseases, Aichi Medical University, Nagakute, Aichi, Japan; ^5^Department of Clinical Infectious Diseases, Kochi Medical School, Nankoku-shi, Kochi, Japan

**Keywords:** *Candida albicans*, *Lactobacillus* species, biofilm, probiotics, cell adhesion

## Abstract

**Introduction:**

Antifungal agents are not always efficient in resolving vulvovaginal candidiasis (VVC), a common genital infection caused by the overgrowth of *Candida* spp., including *Candida albicans*, or in preventing recurrent infections. Although lactobacilli (which are dominant microorganisms constituting healthy human vaginal microbiota) are important barriers against VVC, the *Lactobacillus* metabolite concentration needed to suppress VVC is unknown.

**Methods:**

We quantitatively evaluated *Lactobacillus* metabolite concentrations to determine their effect on *Candida* spp., including 27 vaginal strains of *Lactobacillus crispatus, L. jensenii, L. gasseri, Lacticaseibacillus rhamnosus*, and *Limosilactobacillus vaginalis*, with inhibitory abilities against biofilms of *C. albicans* clinical isolates.

**Results:**

*Lactobacillus* culture supernatants suppressed viable fungi by approximately 24%-92% relative to preformed *C. albicans* biofilms; however, their suppression differed among strains and not species. A moderate negative correlation was found between *Lactobacillus* lactate production and biofilm formation, but no correlation was observed between hydrogen peroxide production and biofilm formation. Both lactate and hydrogen peroxide were required to suppress *C. albicans* planktonic cell growth. *Lactobacillus* strains that significantly inhibited biofilm formation in culture supernatant also inhibited *C. albicans* adhesion to epithelial cells in an actual live bacterial adhesion competition test.

**Discussion:**

Healthy human microflora and their metabolites may play important roles in the development of new antifungal agent against *C. albicans*-induced VVC.

## 1 Introduction

Fungal diseases cause considerable morbidity and mortality, resulting in a high economic burden (Drgona et al., 2014; Bongomin et al., 2017). Vulvovaginal candidiasis (VVC), a common genital infection, is commonly caused by *Candida albicans*, with a lifetime prevalence of up to 78% in women (Yano et al., 2019; Willems et al., 2020). Eight percent of women with VVC experience recurrent VVC (RVVC), which relapses more than four times a year due to the low response to antifungal treatment, including the use of azoles such as fluconazole (Denning et al., 2018; Cooke et al., 2022). The highest prevalence of RVVC occurs among 25-34-year-olds, and it has an annual economic burden of US$14-39 billion in developed countries because it reduces the quality of life (Denning et al., 2018). The emergence and spread of antimicrobial resistance (AMR) have become a global concern, and fungal infections have been excluded from the AMR program (Fisher et al., 2022). However, as with bacterial infections, the use of antifungal drugs is strongly implicated in the occurrence of pathogenic fungi, and thus new methods of prevention or treatment of RVVC that are not dependent on antifungal use are required (Matsubara et al., 2016).

*C. albicans* is a dimorphic fungus that can transform from yeast to an invasive filamentous hyphal form (Sudbery, 2011; Willems et al., 2020). Biofilm formation by *C. albicans* commonly consists of four major stages: yeast cells adhere to a substrate to form a yeast basal layer; initiation of propagating cells where the hyphae are formed; hypha formation and extracellular matrix accumulation with extracellular polysaccharides, structural proteins, cell debris, and nucleic acids; and dispersion of yeast cells from the biofilm to initiate biofilms at new locations (Chandra and Mukherjee, 2015). These biofilm structures are intrinsically resistant to antifungals, making VVC difficult to combat (Silva et al., 2017).

The vaginal microbiota of humans is known to be less complex than the intestinal microbiota and is usually dominated by the genus *Lactobacillus* (Matsumoto et al., 2018). The disruption of this vaginal microbiota promotes colonization by pathogenic microorganisms that leads to bacterial vaginosis and subsequent VVC (Ravel et al., 2013). With recent progress in sequencing technology, the presence of certain lactobacilli has been found to be associated with vaginal health. *Lactobacillus crispatus*-and *L. jensenii*-dominated vaginal microbiota are strongly associated with vaginal health (Chee et al., 2020). Furthermore, a *L. iners*-dominated environment could be affected by vaginal dysbiosis (Chee et al., 2020). These species contribute to vaginal homeostasis mainly by producing metabolites, including lactate and hydrogen peroxide, although their production abilities vary among isolates of the same species (Witkin et al., 2013).

In previous studies, production of lactate and hydrogen peroxide has been evaluated using a qualitative method (Matsubara et al., 2016; Ribeiro et al., 2017; Wang et al., 2017; Aarti et al., 2018; Rossoni et al., 2018). However, studies on quantitative evaluation of the metabolite are insufficient. This study aimed to quantitatively evaluate the metabolites of lactobacilli to determine the effects of lactobacilli on *C. albicans* growth, hyphal formation, biofilm development, and epithelial cell adhesion.

## 2 Materials and methods

### 2.1 Strains

Forty-five *C. albicans* strains, which were clinically isolated from the vagina and provided by Microskylab Inc. (Tokyo, Japan), were used in this study. All 27 *Lactobacillus* strains were previously obtained from vaginal swabs of healthy Japanese women at Aichi Medical University (Matsumoto et al., 2018). These strains belonged to five species: *L. crispatus*, *L. jensenii*, *L. gasseri*, *Lacticaseibacillus rhamnosus*, and *Limosilactobacillus vaginalis*. The characteristics of these bacterial strains are listed in **Supplementary Table 1**.

### 2.2 Biofilm formation and quantification

A 96-well microtiter plate-based method was used in this study (Lohse et al., 2018). *C. albicans* strains were cultured for 24 h in Yeast Peptone Dextrose (YPD) agar (Difco, Detroit, MI, USA) at 30°C under aerobic conditions. A single colony was inoculated into the YPD broth medium and incubated overnight for 16 h at 30°C, accompanied with shaking at 160 rpm under aerobic conditions. Under these conditions, *C. albicans* strains grew to the budding yeast forms (blastospores). The cells were centrifuged at 3,500 ×g for 10 min and re-suspended in RPMI 1640 medium buffered with morpholinepropanesulfonic acid (MOPS) at a concentration of 10^7^ cells/mL, and 100 μL of the inoculum was seeded into a 96-well microtiter plate. The biofilms formed on the surface of the wells were gently washed twice with phosphate-buffered saline (PBS) after 48 h of incubation at 37°C. The yeast cells were not washed immediately after the initial adhesion, and thus, the final time point (48 h) reflects the total biomass that could not be initially adhered to. Crystal violet (CV) (Merck KGaA, Darmstadt, Germany) and water-soluble tetrazolium salts (WST-1) (TaKaRa, Shiga, Japan) were used in this study (Mukherjee et al., 2005; Weber et al., 2008). CV stained the whole biomass, including dead cells and polysaccharides, whereas WST was converted to a colored formazan in the presence of metabolic activity. To quantify the total biomass, washed biofilms were stained with 0.1% (w/v) CV solution for 1 min. Each well was washed twice with PBS and dried for 30 min. The bounded CV was eluted using 99.5% (v/v) ethanol. The burden of viable cells was estimated using WST-1 based on the reduction of tetrazolium salt. To each well, we added 100 μL of PBS and 10 μL of premixed WST-1, and the mixture was incubated at 37°C for 3 h under shade conditions. The absorbance (Abs) of CV and WST-1 was measured at 570 nm and 440 nm, respectively. Eight replicate wells were used for each strain, and experiments was repeated three times, independently.

### 2.3 Quantitative reverse transcription polymerase chain reaction

ISOGEN II (Nippon Gene, Co., Ltd., Tokyo, Japan) was used for total RNA extraction from the *C. albicans* HB-10 strain. The RNA concentrations were measured using a Qubit^®^ RNA Assay Kit (Promega, WI, USA). To prepare complementary DNA, the PrimeScript™ RT reagent kit (TaKaRa, Shiga, Japan) was used in accordance with the manufacturer’s instructions. Moreover, qRT-PCR analysis was performed using TB Green^®^ Premix Ex Taq™ II (Tli RNaseH Plus) (TaKaRa, Shiga, Japan) in accordance with the manufacturer’s protocol. Briefly, PCR was performed in a reaction mixture of TB Green Premix Ex Taq II (2 ×) 12.5 µL, PCR forward primer 1 µL, PCR reverse primer 1 µL, and RNase free dH_2_O 8.5 µL added to 2 µL of each reverse transcription reaction solution. Primers used in this study are listed in **Supplementary Table 2**. The amplification conditions were as follows: 40 cycles under heat treatment at 95°C for 30 s, heat denaturation at 95°C for 5 s, and annealing at 55°C for 30 s, which is the optimum temperature for the primer. Melting curves were used to verify the quality of qRT-PCR, and the fold expression was calculated using the delta-delta Ct method.

### 2.4 Supernatants produced by *Lactobacillus*

Cell-free culture supernatants were extracted from *Lactobacillus* species. A single strain each was inoculated in de Man, Rogosa, and Sharpe (MRS) broth (Merck KGaA, Darmstadt, Germany) and incubated at 37°C for 72-h under anaerobic conditions (10% H_2_, 10% CO_2_, and 80% N_2_) in an anaerobic chamber. Growth at the sampling point (72-h) was determined by measuring the optical density (OD) at 600 nm using a microplate reader (SH-9000Lab, HITACHI). The culture medium was then centrifuged at 3,500 ×*g* for 10 min and filtered through a 0.22-μm membrane filter (Sarutorius AG, Gettingen, Germany). Each collected culture supernatant was stored at −80°C until use.

### 2.5 High-performance liquid chromatography analysis of culture supernatants

HPLC (SHIMADZU, Kyoto, Japan) equipped with a conductivity detector was used to measure levels of lactate and short-chain fatty acids, such as acetate, propionate, and butyrate, in culture supernatants, as previously described (Hagihara et al., 2021). Briefly, the mobile phase required 5 mM p-toluenesulfonic acid (KANTO Chemical, Tokyo, Japan). The reaction buffer was made of 5 mM p-toluenesulfonic acid, 100 μM ethylenediaminetetraacetic acid (KANTO Chemical, Tokyo, Japan), and 20 mM bis (2-hydroxyethyl) aminotris (hydroxymethyl) methane (Tokyo Chemical Industry, Tokyo, Japan). The flow rate, oven temperature, and detector cell temperature were set at 0.8 mL/min, 40°C, and 48°C, respectively. The samples contained in 1.0 mL disposable vials (SHIMADZU Co., Kyoto, Japan) were held at 4°C in a sample cooler (SHIMADZU, Kyoto, Japan), and 10 μL was applied to tandemly arranged two columns (SHIMADZU, Kyoto, Japan) to measure lactate levels. The calibration curve solution adjusted with lithium DL-lactate (FUJIFILM Wako Pure Chemical, Co., Ltd., Osaka, Japan) was dissolved in deionized water. The quantification analyses for HPLC were performed using LabSolutions version 5.90 (SHIMADZU Co., Kyoto, Japan), and the peak area was used as the signal intensity.

### 2.6 Detection of hydrogen peroxide in culture supernatants

Hydrogen peroxide production was estimated using a hydrogen peroxide assay kit (ab102500, Abcam, MA, USA) according to the manufacturer’s instructions. The stored culture supernatant was neutralized to pH 7.0, and 100 μL of the adjusted supernatants were reacted for 10 min in the presence of horseradish peroxidase. Duplicate wells were measured for each sample with absorbance at 595 nm, and experiments were repeated three times, independently.

### 2.7 Detection of pH in culture supernatants

The pH of cell-free culture supernatants and buffered RPMI 1640 medium, supplemented with culture supernatant, were measured promptly using a glass electrode-style hydrogen-ion concentration meter (Laqua, Horiba, Ltd., Japan). MRS (8%) was added to RPMI instead of *Lactobacillus* supernatant to achieve final concentrations of lactate and hydrogen peroxide standard of 4–64 mM and 4 nM–64 nM, respectively.

### 2.8 Measurement of minimum inhibitory concentration

*C. albicans* sHB-10 were cultured for 24 h in YPD agar at 30°C under aerobic conditions. A single colony was inoculated into the YPD broth medium and incubated overnight for 16 h at 30°C, accompanied with shaking at 160 rpm under aerobic conditions. The cells were centrifuged at 3,500 ×g for 10 min and re-suspended in RPMI 1640 medium at a concentration of 10^6^ cells/mL. Lactate and hydrogen peroxide were added to the RPMI broth with 8% MRS and adjusted to a concentration ranging from 0.5 to 1024 mM and 0.5 nM to 1024 mM, respectively. After 24 hours of incubation at 37°C, the turbidity of all well broth was visually observed, and the lowest concentration of lactate or hydrogen peroxide that suppressed the increased growth was determined as the MIC.

### 2.9 Effect of *Lactobacillus* culture supernatants on preformed *C. albicans* biofilm

The efficacy of the anti-biofilm activities of lactobacilli was determined by adding the culture supernatant of *Lactobacillus* to the preformed biofilm. *C. albicans* HB-10, which formed a mature biofilm in the assay described above, was selected and used for subsequent inhibition assays. A mature biofilm of *C. albicans* HB-10 was formed in a 96-well microtiter plate after 24 h of incubation under the same conditions as the biofilm formation and viability assay. The planktonic cells were aspirated from each well and washed twice with PBS. Cell-free supernatant extracted from a single *Lactobacillus* strain was added to each well at a final concentration of 8% (v/v) and incubated for 24 h. Culture supernatants were aspirated from each well and washed twice with PBS. Biofilm formation was quantified using CV and WST-1, as described above. The metabolic activity of the residual biofilm after the exposure of the *Lactobacillus* culture supernatant was quantified using WST-1 as described above. Eight replicate wells were used for each strains, and experiments was repeated three times, independently.

### 2.10 Effect of *Lactobacillus* culture supernatants on *C. albicans* hyphal formation

According to the hyphal formation method in the RPMI broth described previously (Wang et al., 2017), we estimated the effect of hyphal formation inhibition of *C. albicans* yeast-to-hyphal transition in the presence of *Lactobacillus* culture supernatants. *Lactobacilli* with strong inhibition of biofilm formation and those with low inhibition were selected. *C. albicans* HB-10 cells from overnight culture were washed with PBS and re-suspended at approximately 10^6^ CFU/mL in RPMI 1640 medium buffered with MOPS. The yeast cell suspensions were then incubated with or without *Lactobacillus* culture supernatant at 37°C for 3 h. Quantification of the inhibitory effect of *Lactobacillus* on hyphal formation was performed using a light microscope (AxioCam MRc5; Carl Zeiss, Jena, Germany). The percentage of hyphal formation was calculated by obtaining the ratio of total number of *C. albicans* cells with hyphae to the total number of *C. albicans* cells counted. The number of yeast and hyphae (total cells) was counted using a hemocytometer (**Supplementary Table 3**).

### 2.11 Adhesion assay of *C. albicans* and *Lactobacilli*

Human cervical cancer HeLa cells (RCB0007; Riken BRC Cell Bank, similar to ATCC CCL2) were grown in Dulbecco’s modified Eagle’s medium (DMEM; Thermo Fisher Scientific, MA, USA) supplemented with 10% (v/v) fetal bovine serum (Thermo Fisher Scientific, MA, USA) and 1% (v/v) penicillin and streptomycin (FUJIFULM Wako Pure Chemical, Co., Ltd., Osaka, Japan) at 37°C under 5% CO_2_ and humidity. HeLa cells were seeded into a 12-well plate (AGC Techno Glass Co., Ltd., Shizuoka, Japan) at approximately 1.0×10^5^ cells per well and grown to confluence. After 90% confluency, each well was washed twice with PBS. *C. albicans* HB-10 and lactobacilli were grown under the conditions described above. Briefly, a single colony of *C. albicans* HB-10 and lactobacilli was inoculated into YPD and MRS broth, respectively. After 48 h of incubation, both *C. albicans* HB-10 and lactobacilli cells were collected using centrifugation at 3,500 ×*g* for 10 min and re-suspended in DMEM. The suspension of *C. albicans* HB-10 and lactobacilli contained approximately 1.0×10^7^ CFU/mL. To the HeLa cell culture well, 100 μL of 10-fold serial dilutions of lactobacilli suspensions was added and incubated at 37°C for 1 h under 5% CO_2_. Subsequently, 100 μL of 10-fold serial dilutions of *C. albicans* HB-10 suspensions was added to each well and incubated for 1 h under the same conditions to allow *C. albicans* HB-10 to adhere to cells. After incubation, each well was washed twice with PBS to remove non-adherent *C. albicans* cells and then treated with 0.05% trypsin-EDTA (Nacalai Tesque, Inc., Kyoto, Japan). The inhibitory rate of adhesion was calculated as the number of *C. albicans* cells that adhered to HeLa cells with *Lactobacillus* pre-treatment, per the number of *C. albicans* cells that adhered to HeLa cells in the absence of lactobacilli.

### 2.12 Statistical analysis

Statistical analyses were performed using R and RStudio (versions 4.0.3 and 1.4.1106, respectively). Mann–Whitney U-test was used to determine significant differences between DMEM control and different lactobacilli. One-way analysis of variance was used to compare multiple groups. Statistical significance was set at *p* values <0.05. Correlations between growth and metabolites (lactate and hydrogen peroxide) were determined using Spearman’s rank correlation coefficient.

## 3 Results

### 3.1 Biofilm formation abilities of *C. albicans*

**Figure 1:**
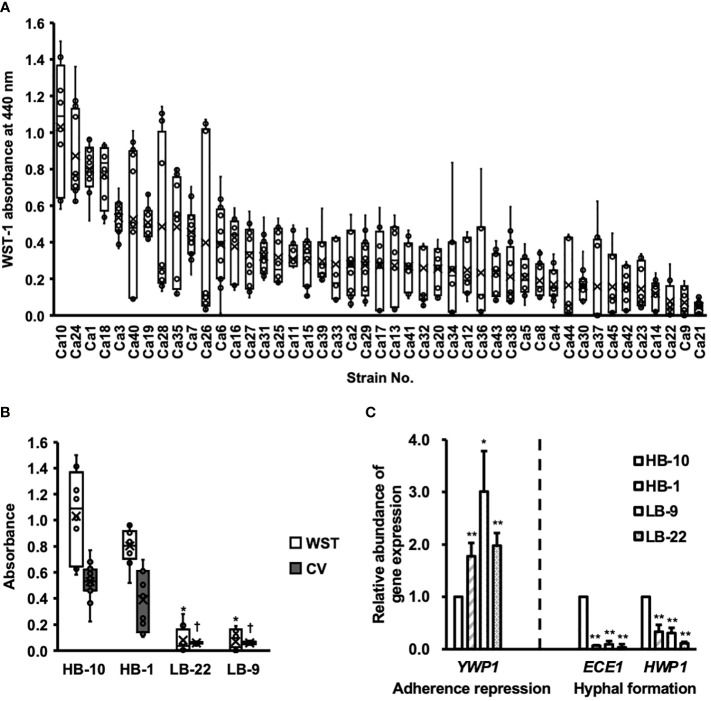
Clinical isolates of *Candida albicans* with different abilities to form biofilms. CV, crystal violet; WST, water-soluble tetrazolium salts. **(A)** The biofilm formation of 45 clinical isolates of *C albicans* were used to measure WST-1 and are exhibited by the box whisker plots. Box plot shows the median (horizontal thick blank line), mean (cross), and first and third quartiles (box). **(B)** Biofilm formation by different *C albicans* strains was estimated using both WST-1 reduction and CV staining. *p < 0.05 by U-test compared with WST-1 absorbance of *C albicans* HB-10. ^†^*p* < 0.05 by U-test compared with crystal violet (CV) absorbance of *C albicans* HB-10. **(C)** Relative quantitation of genes associated with adherence repression (*YWP1*), or hyphal formation (*HWP1* and *ECE1*) normalized to the β-actin gene. The *C albicans* HB-10 strain was used as the reference to depict the difference among the four *C albicans* clinical isolates. Bars represent the standard deviation from the mean values. *p < 0.05 and **p < 0.01 by U-test.

Biofilm formation by *C. albicans* clinical isolates was assessed using the WST-1 formazan dye (**Figure 1A**).

Among these 45 strains, representative strains that reproduced well and showed significant differences in the CV assay were selected as high and low biofilm-producing strains and renamed as *C. albicans* HB-1 and HB-10 and *C. albicans* LB-9 and LB-22, respectively (**Figure 1B**). The expression levels of the three genes, *ECE1*, *HWP1*, and *YWP1*, which regulate different stages of biofilm formation in *C. albicans*, were determined using qRT-PCR (**Figure 1C**).

Four *C. albicans* strains with gene expression of *HWP1*, *ECE1*, *and YWP1* exhibited bar plots normalized by *C. albicans* HB-10 gene expression levels. These gene expression levels were calculated according to *ACT1* gene expression levels. Similar to the phenotypic biofilm-forming analysis, the relative gene expression levels of *ECE1* and *HWP1* in the HB-10 strain were significantly higher than those in the LB-9 (10.53-fold and 3.21-fold, respectively) and LB-22 strains (31.21-fold and 8.72-fold, respectively) (*p* < 0.05). Interestingly, the HB-1 strain, which was a high biofilm producer and did not show such a large difference in biofilm-forming ability, had significantly lower expression levels of these genes than the HB-10 strain. In contrast, *YWP1*, which suppressed initial adhesion, was the lowest in the HB-10 strain.

### 3.2 Characterization of culture supernatant

**Figure 2:**
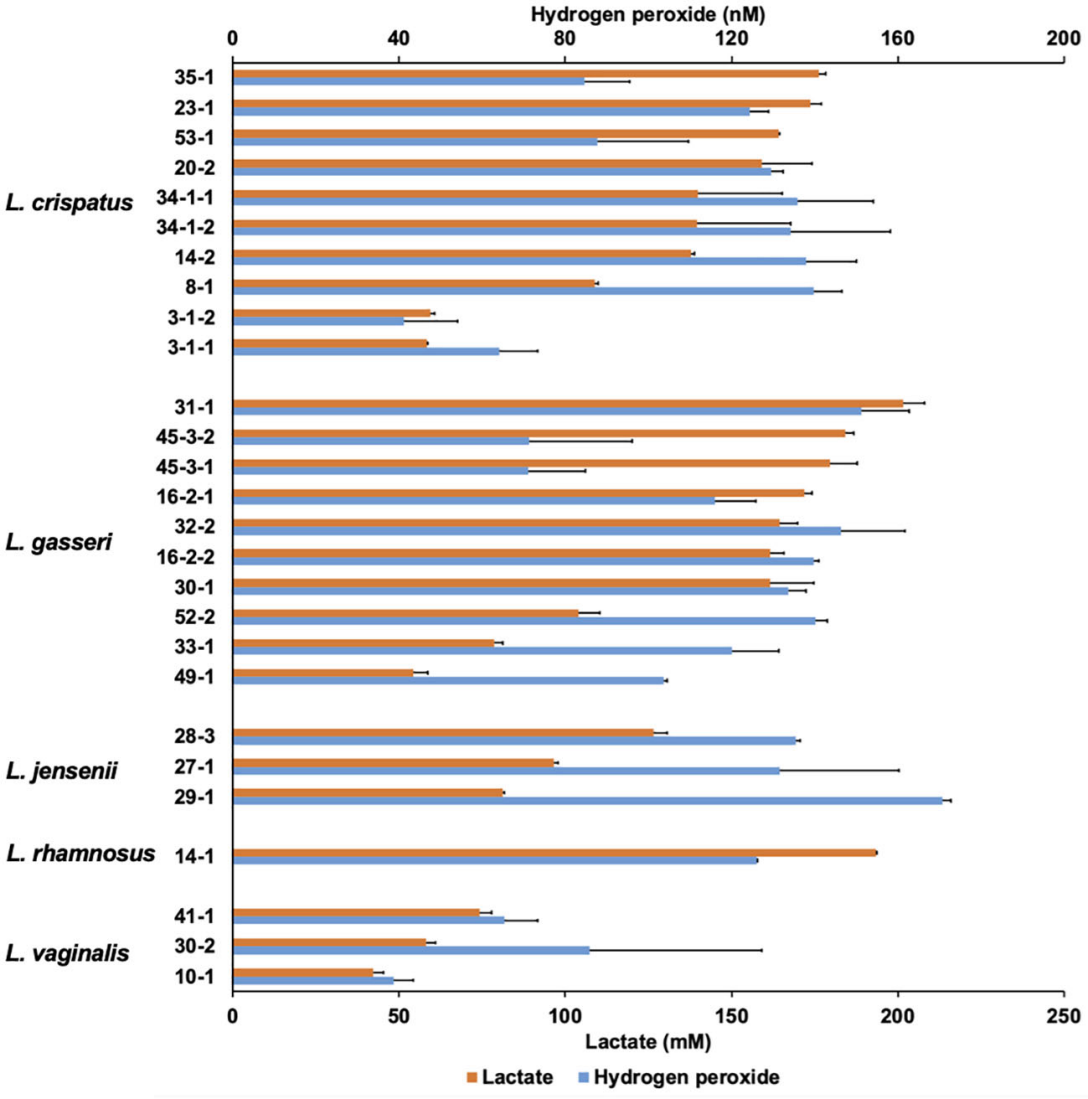
Lactate and hydrogen peroxide production by 27 *Lactobacillus* clinical isolates. Twenty-seven *Lactobacillus* clinical isolates were cultured in de Man, Rogosa, and Sharpe (MRS) broth for 72 h, and cell-free culture supernatants were collected. Lactate level was measured quantitatively by high-performance liquid chromatography (HPLC), and hydrogen peroxide was measured quantitatively using a hydrogen peroxide assay kit. Data are represented by the mean across the three replicates.

Vaginal lactobacilli produce various metabolites that exhibit antifungal activity. Cell-free culture supernatants extracted from 27 strains of *Lactobacillus* belonging to five species after 48-h incubation were characterized (**Figure 2**).

The lactate and hydrogen peroxide production of lactobacilli used in this study ranged from 42.1 to 201.7 mM and 38.7 to 170.8 nM, respectively. There was no correlation between lactate production and hydrogen peroxide production (*r* = 0.426; *p* = 0.217). In contrast, OD corresponding to the growth of *Lactobacilli* and lactate levels at the 72-h sampling point showed moderately positive correlations (*r* = 0.667; *p* < 0.001), while OD and hydrogen peroxide levels showed no correlation (*r* = 0.265; *p* = 0.181) (**Supplementary Figure 2**). In *L. jensenii*, the average hydrogen peroxide production was the highest compared to other species (mean 146.0 nM), although lactate production was not as high (ranging from 81.3 to 126.5 mM).

### 3.3 Effect of *Lactobacillus* culture supernatant on preformed biofilm

**Figure 3:**
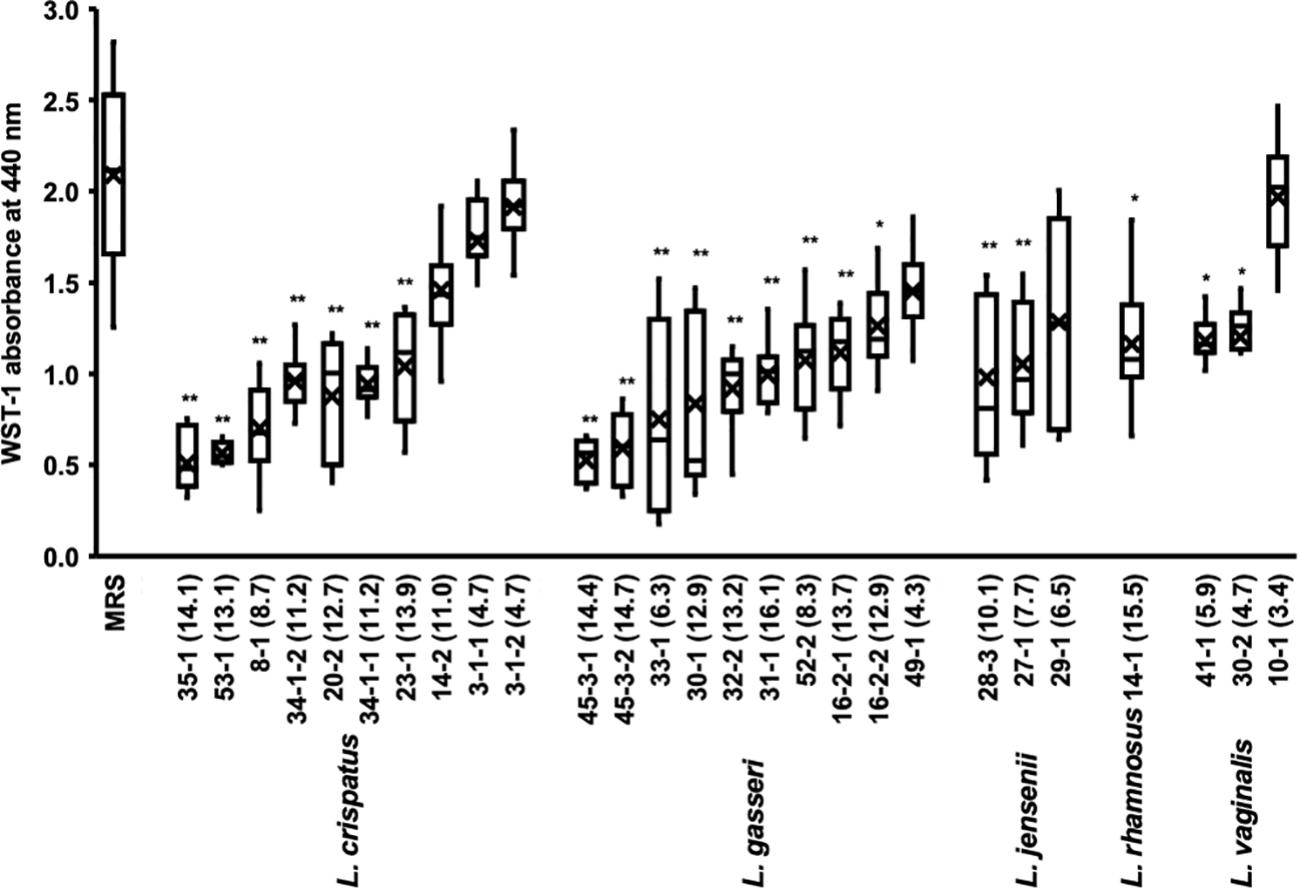
Metabolic activity of the biofilm of *C. albicans* HB-10 treated with culture supernatants of 27 different *Lactobacillus* clinical isolates. The x-axis indicates the strain number and lactic acid concentration (mM). The burden of viable cells of preformed biofilm treated after culture supernatants of 27 different *Lactobacillus* clinical isolates was measured using the WST-1 reduction reaction. MRS broth was used as the control. WST, water-soluble tetrazolium salts; MRS, de Man, Rogosa, and Sharpe. Box plot shows the median (horizontal thick blank line), mean (cross), and first and third quartiles (box). Bars represent the standard deviation from the mean values. *p < 0.05 and **p < 0.01 by U-test.

In a typical experiment using 96 well plates, biofilm formation takes 24 h to reach confluency. We investigated the effects of *Lactobacillus* culture supernatants on preformed biofilms (**Figure 3**).

The addition of *Lactobacillus* culture supernatant resulted in 24.3%-91.8% relative WST-1 readings compared with those of non-added control. The culture supernatants of *L. crispatus*, *L. gasseri*, *L. jensenii*, and *L. vaginalis* with average relative WST-1 readings were approximately 52.5%, 43.1%, 57.2%, and 58.9%, respectively. The effect of different lactobacilli species on preformed biofilms of *C. albicans* HB-10 did not differ significantly. A moderate negative correlation was found between *Lactobacillus* lactate production and WST-1 readings (*r* = −0.625; *p <*0.001), but no correlation was observed between hydrogen peroxide production and WST-1 readings (**Supplementary Figure 3**).

We added several concentrations of the standards to the biofilm to reproduce lactate and hydrogen peroxide as metabolites in the culture supernatant (**Supplementary Figure 4**). The results showed that lactate concentrations had lower WST values than controls at all concentrations except 4 mM and a concentration-dependent effect on WST values (*r* = -0.930; *p* = 0.001). In contrast, hydrogen peroxide had no concentration-dependent effect on the preformed biofilm, with WST values not significantly different from the control at all concentrations. Lactate and hydrogen peroxide further showed no additive or synergistic effects on the preformed biofilms. A strong effect on the preformed biofilm was observed when the culture supernatant of the *L. crispatus* 35-1 strain was added (final concentration 14.1 mM), which was consistent with the WST-1 values for the biofilm when the 16 mM lactate standard was added (**Figure 3** and **Supplementary Figure 4**).

### 3.4 Effect of *Lactobacillus* culture supernatant on the growth of planktonic cultures

The inhibitory effect of *Lactobacillus* spp. on *C. albicans* yeast cell growth was also evaluated. Significant growth inhibition was shown in 4/27 (14.8%) of the strains with the addition of culture supernatant of each *Lactobacillus* as follows: *L. crispatus* strain 23-1 and 20-2, *L. gasseri* strain 31-1 and *L. rhamnosus* strain 14-1. Interestingly, three of these strains showed lactate and hydrogen peroxide production above 165 mM and 120 nM, respectively (**Supplementary Figure 7**). The MICs for lactate and hydrogen peroxide standard for *C. albicans* HB-10 samples were 512 mM and 20 mM, respectively.

### 3.5 Effect of *Lactobacillus* culture supernatant on the hyphal formation

**Figure 4:**
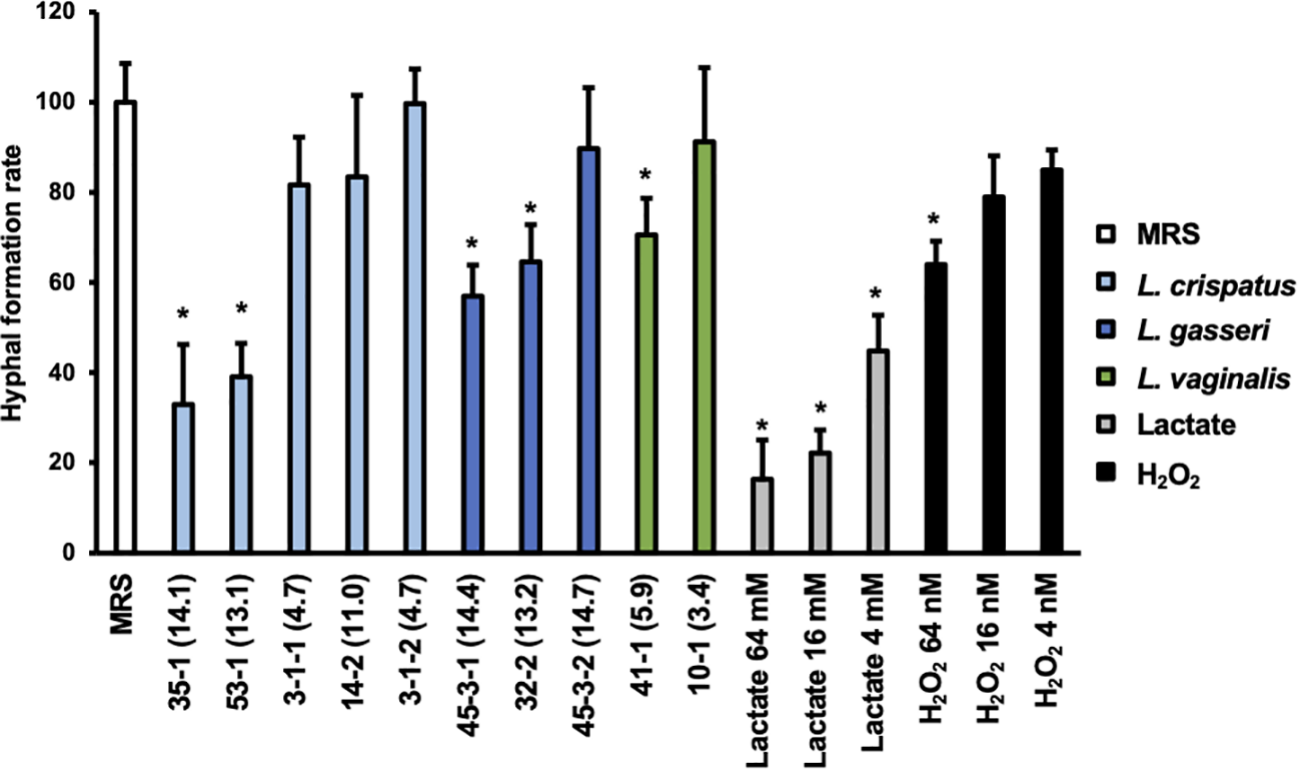
Hyphal formation rate of *C. albicans* HB-10 strain treated with *Lactobacillus* culture supernatants, lactate, or hydrogen peroxide. The x-axis indicates the strain number and lactic acid concentration (mM) or lactate concentration or hydrogen peroxide concentration. Relative hyphal formation in *C. albicans* HB-10 treated with culture supernatants of 10 different *Lactobacillus* clinical isolates, lactate, or hydrogen peroxide. Bars represent the standard deviation from the mean values. MRS broth was used as the control. MRS, de Man, Rogosa, and Sharpe. *p < 0.05 by U-test.

The effect on the rate of hyphal formation was compared for *Lactobacillus* culture supernatants that exhibited significant differences in their WST values to the preformed biofilm and for those that did not (**Figure 4**).

Yeast, hyphae, and pseudohyphae were identified under a microscope (**Supplementary Figure 8**). The addition of MRS medium control resulted in 54.95 ± 9.61% of the hyphae, and pseudohyphae were identified after 3 h of incubation. Compared to MRS control alone, lactate standards showed a concentration-dependent decrease in hyphal formation at 16 to 64 mM (*p*<0.05), while only 64 nM of hydrogen peroxide showed a significant difference. The percentage of hyphal formation by *Lactobacillus* culture supernatants ranged from 18.11 to 54.77%. In terms of the percentage of a hyphal formation relative to untreated MRS, significant decreases were observed with the addition of culture supernatant in *L. crispatus* strain 35-1 (32.97 ± 13.29%) and 53-1 (39.10 ± 7.40%), *L. gasseri* strain 45-3-1 (56.99 ± 6.90%) and 32-2 (70.75 ± 4.15%), and *L. vaginalis* strain 41-1 (70.60 ± 8.12%). All of these showed final lactate concentrations >50 mM. In contrast, although there were no differences in lactate and hydrogen peroxide metabolite profiles between *L. gasseri* strains 45-3-1 and 45-3-2, only strain 45-3-1 showed significantly lower hyphal formation than the MRS control.

### 3.6 Effect of *Lactobacillus* bacterial cell on the initial adhesion

**Figure 5:**
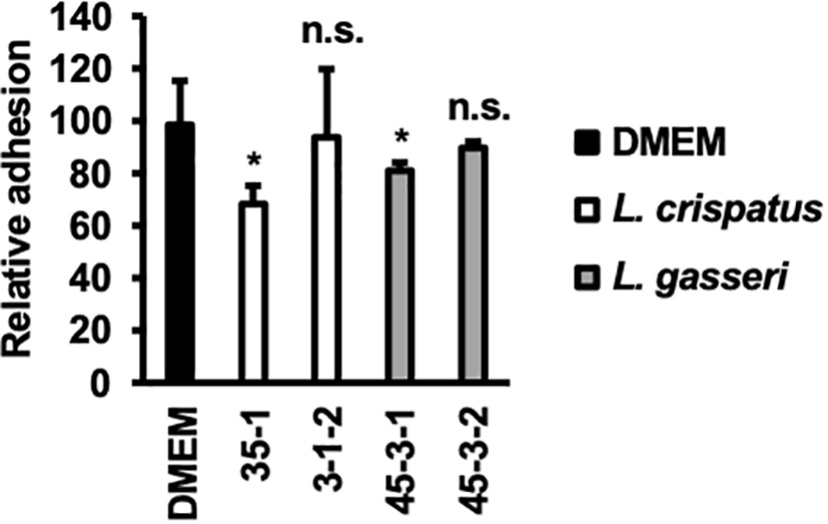
Adhesion of *C. albicans* HB-10 strain to HeLa cells according to the presence or absence of lactobacilli. Relative adherence of the *C. albicans* HB-10 strain to HeLa cells pretreated with DMEM or different lactobacilli. Bars represent the standard deviation from the mean values. *p < 0.05 by U-test. DMEM, Dulbecco’s modified Eagle’s medium. n.s., not significant.

The inhibition of *C. albicans* yeast adhesion to human epithelial cells by *Lactobacillus* bacterial cells was assessed (**Figure 5**).

*L. crispatus* strain 35-1 showed a more efficient inhibition of *C. albicans* HB-10 (adhesion rate: 68.29 ± 6.90%). In contrast, *L. crispatus* strain 3-1-2 showed no statistically significant difference (adhesion rate: 93.90 ± 25.87%). Interestingly, *L. gasseri* strain 45-3-1, which showed inhibition of hyphal formation, also showed a significant reduction in *C. albicans* HB-10 adhesion (80.95 ± 3.17%), whereas strain 45-3-2 showed no inhibitory effect on initial adhesion (89.68 ± 2.38%).

## 4 Discussion

This study presents the steps of *C. albicans* biofilm formation that are affected by clinical isolates of lactobacilli. The analysis focuses on lactate and hydrogen peroxide among the metabolites (culture supernatants), and adhesion analyses were performed using viable bacteria.

The virulence of *C. albicans* in VVC is complexly related to multiple factors such as adhesion to cell surfaces and inert surfaces, cell damage by hydrolases and candidalysin, and subsequent active hyphal invasion, biofilm formation, and phenotypic switching (Berman and Sudbery, 2002; Moyes et al., 2016; Czechowicz et al., 2022). *C. albicans* yeast cells express adhesin and adhere to the host cell surface. HWP1 and a group of eight glycosylated proteins (*ALS1-ALS7* and *ALS9*) associated with the ALS gene are important adhesins (Rodríguez-Cerdeira et al., 2020). HWP1 is important as a component of the hyphal cell wall and may stabilize biofilms by adhering to yeast cells and hyphae in biofilms, making them highly pathogenic to the host (Zhu and Filler, 2010; Talapko et al., 2021). In addition to adhesion to cell surfaces, HWP1 is involved in adhesion to inert surfaces (Nobile et al., 2006). *C. albicans* yeast cells are transferred to hyphae by various environmental factors such as pH, CO_2_ concentration, temperature, and N-acetylglucosamine (Sudbery, 2011). *C. albicans* invades cells from the cell surface in two ways: passive invasion by endocytosis and active invasion by disrupting the cell surface with hydrolases and candidalysin (Moyes et al., 2016; Maza et al., 2017). In particular, candidalysin, encoded by the ECE1 gene, directly disrupts epithelial cells by acting as a cytolytic peptide toxin (Moyes et al., 2016). *C. albicans s*ecretes candidalysin into the hyphal entry pocket, effectively destroying the tissue and establishing a mucosal infection with *C. albicans* (Mogavero et al., 2021). YWP1 inhibits adhesion of *C. albicans* yeast cells to the cell surface. Furthermore, YWP1 may express mannoproteins on the outer layer of the yeast cell wall, which may cover the epitope β-1,3-glucan and allow it to escape the immune system (Granger, 2012; Granger, 2018).

In this study, *C. albicans* biofilms are formed on inert surfaces (microtiter plates). For this reason, we evaluated the expression level of the HWP1 gene, which is important for inert surface attachment and is also associated with hyphal formation (Nobile et al., 2006). Candidalysin is encoded by *ECE1* and is important for active invasion of *C. albicans* by disrupting the host cell surface (Moyes et al., 2016). Since *ECE1* is an important gene for the invasion of C. albicans hyphae into HeLa cells, which are biotic surfaces, we evaluated the gene expression of *ECE1*. HB-10, which formed the highest amount of biofilms on inert surfaces (microtiter plates), was found to express high levels of HWP1. In contrast, HB-1 formed high biofilms, although the gene expression levels of *HWP1* and *ECE1* were low, indicating a dissociation between phenotype and gene expression. This may be because hyphal formation and invasion of epithelial cell, which is important for biofilm formation, are composed of multiple signal transduction pathways (Sudbery, 2011). For the *C. albicans* HB-10 biofilm, *C. albicans* biofilm formations were initiated using a 96-well plate in this study.

A strong effect on the preformed biofilm was observed when the culture supernatant of the *L. crispatus* 35-1 strain was added (final concentration, 14.1 mM), which is consistent with the WST-1 values for the biofilm when the 16 mM lactate standard was added. A healthy human vaginal environment is maintained at a low pH. In this acidic pH environment, *C. albicans* is less likely to undergo a morphological yeast-fungus transition (Davis et al., 2000). The pH of the buffered RPMI 1640 medium used in this study, supplemented with culture supernatant of the *L. crispatus* 35-1 strain (final concentration 14.1 mM) and 16 mM lactate standard, had similar levels (at a pH range of 4.3 to 4.6) This suggests that direct pH reduction due to lactate might be responsible for the anti-*C. albicans* activities. This is confirmed by the fact that the inhibition of biofilm and hyphal formation disappears when the 4–64 mM Lactate standard is neutralized using NaOH (**Supplementary Figures 5**, **6**). The MIC values for planktonic yeast were 512 mM for lactate and 20 mM for hydrogen peroxide. For the preformed biofilm in **Figure 3**, the concentration range for 8% *Lactobacillus* supernatant addition was 3.4 - 16.1 mM for lactate and 3.1 - 13.7 nM for hydrogen peroxide. In **Supplementary Figure 6**, regarding lactate and hydrogen peroxide standard samples, lactate inhibited preformed biofilms at 16 mM-64 mM; however, hydrogen peroxide did not inhibit at all in any concentration. Therefore, at sub-MIC concentrations, lactate (both supernatant and standard samples) may show fungistatic activities, whereas hydrogen peroxide may not. Fluconazole, a therapeutic agent for VVC, shows fungistatic activities against *C. albicans*, and increased susceptibility to fluconazole has been reported in biofilms in the presence of lactate (Alves et al., 2017). This could lead to the development of a new *C. albicans* treatment by combining *Lactobacillus* and fluconazole.

Hyphal formation and growth are associated with *C. albicans* virulence (Jang et al., 2019; Roselletti et al., 2019). HB-10 used in this study expresses the *ECE1* gene; Ece1p, a protease encoded by *ECE1*, causes inflammation in epithelial cells and allows *C. albicans* hyphae to adhere to and invade the cell epithelium (Moyes et al., 2016). To inhibit biofilm formation, it is important to prevent *C. albicans* adhesion to the epithelial cells. In the present study, lactate inhibited hyphal formation in a concentration-dependent manner. Despite the similar metabolic profiles of lactate and hydrogen peroxide in *L. gasseri* strains 45-3-1 and 45-3-2, only strain 45-3-1 significantly inhibited hyphal formation. Similarly, among the *L. gasseri* strains 45-3-1 and 45-3-2, only strain 45-3-1 significantly inhibited *C. albicans* adhesion to epithelial cells. This suggests that metabolites other than lactate and hydrogen peroxide inhibit hyphal formation. Indeed, recent studies have suggested that small molecules produced by *Lactobacillus* may inhibit *C. albicans* biofilm formation and growth as antimicrobial compounds (Lee et al., 2021; MacAlpine et al., 2021). *L. crispatus* strain 35-1 and *L. gasseri* strain 45-3-1 showed a significant reduction in *C. albicans* HB-10 adhesion (adhesion rates: 68.29 ± 6.90% and 80.95 ± 3.17%, respectively) to HeLa cells. Thus, different *Lactobacillus* strains showed different rates of inhibition of *C. albicans* HB-10 adhesion to HeLa cells. In the experimental setup of this study, *Lactobacillus* first adhered to HeLa cells before *C. albicans* was added. Although an accurate count of lactobacilli, which adhere to HeLa cells could not be obtained in this study, our findings reveal the *Lactobacillus* strain with high attachment ability to HeLa cells, which may preferentially adhere to a limited number of epithelial cell surfaces, indicating that *C. albicans* was physically unable to adhere to these cells. In this study, *L. crispatus* 35-1 and *L. gasseri* 45-3-1 strains inhibited *C. albicans* adhesion but failed to reduce it to less than 50%. Thus, it should be noted that in terms of multiplicity of infection, the inhibitory effect of *Lactobacillus* used in this study on *C. albicans*’s epithelial cell attachment is not so strong.

Several studies using clinical isolates and deposited strains in biofilm formation inhibition testing have been reported. Culture supernatant of a clinically isolated strain, *Lactobacillus crispatus* BC1-BC8, inhibited biofilm formation (Itapary Dos Santos et al., 2019). Compared to no cell-free culture supernatants, culture supernatants of deposited strains, *Lactobacillus fermentum* ATCC 23271 and *L. rhamnosus* ATCC 9595, inhibited biofilm formation by more than 40% in the CV and 2,3-bis-(2-methoxy-4-nitro-5-sulfophenyl)-2H-tetrazolium-5-carboxanilide assays (Itapary Dos Santos et al., 2019). On the other hand, the addition of cell-free culture supernatants of *Lactobacillus* iners ATCC 55195 significantly increased hyphal and biofilm formation of *C. albicans* compared to the control (Sabbatini et al., 2021). Matsuda et al. reported no inhibitory effect when 7.5% *L. crispatus* JCM 1185 and *L. gasseri* JCM 1131 culture supernatants were added to the *C. albicans* preformed biofilm (Matsuda et al., 2018). In our study, culture supernatants of 8% *L. crispatus* JCM 1185 and *L. gasseri* JCM 1131 exhibited no inhibitory effect on the biofilm (Residual biofilm 78.2% and 66.7% compared with no cell-free culture supernatants control), suggesting that the reproducibility of previous reports has been achieved. However, *L. crispatus* 35 -1 and *L. gasseri* 45 -3 -1 in this study significantly inhibited the pre-formed biofilm. The hyphae formation rate of *L. crispatus* JCM 1185 was 28.0%, which was not significantly different from that of clinical isolates. In contrast, that of *L. gasseri* JCM 1131 was 6.6%, which is interesting because it has a higher inhibitory effect than clinical isolates.

A worrisome trend is that VVC caused by non-*albicans Candida* species (NAC), *C. tropicalis, C. krusei*, and *C. glabrata*, has been increasing (Zhou et al., 2010; Ravel et al., 2011). In particular, *C. tropicalis* is frequently isolated in Asia and is known to have high hyphal budding ability and form strong biofilms that are resistant to treatment (Bizerra et al., 2008; Ravel et al., 2011; Kawai et al., 2017). Although *C. tropicalis* has good *in vitro* drug susceptibility to azoles, candins, and polyenes, the poor clinical prognosis may be related to biofilm formation (Yamagishi et al., 2009; Sakagami et al., 2019). Visualization of biofilm formation has shown that candin- and polyene-based drugs are suitable for biofilm-forming NAC (Kawai et al., 2015; Kawai et al., 2017). In the actual human vaginal environment, glycogen is digested by α-amylase to produce maltose, maltotriose, and maltotetraose (Spear et al., 2014). *Lactobacilli* are known to consume glycogen-breakdown products to produce lactate. However, *in vitro* experiments have not fully mimicked the vaginal environment with respect to nutrient sources for *Lactobacillus* development, which may have affected their growth and metabolite production (Spear et al., 2014; Nunn et al., 2020). Thus, classically defined bacterial aerobes and anaerobes form a community of microaerophilic environments in the mucosa lining the vaginal lumen. In this study, optimal growth environments for *Lactobacillus* and *C. albicans* were selected (anaerobic and aerobic conditions, respectively). However, it is difficult to reproduce the complex vaginal ecosystem under a single culture condition in an *in vitro* experimental system; thus, it is necessary to set *Lactobacillus* and *C. albicans* in aerobic, microaerobic, and anaerobic conditions to evaluate biofilms. The results of this study suggest that *Lactobacillus* metabolites other than lactate and hydrogen peroxide may also affect *C. albicans*, although they have not been evaluated in detail. In future, the effects of various metabolites produced by *Lactobacillus* on *C. albicans* need to be evaluated under conditions that are more similar to the human vaginal environment. In this study, the effects of lactate and hydrogen peroxide on *C. albicans* HB10 biofilm and hyphal formation are investigated using lactate and hydrogen peroxide standard samples. However, metabolites other than lactate and hydrogen peroxide are possibly involved in *C. albicans* biofilm formation. Therefore, it is necessary to consider the effects of the absence of lactate and hydrogen peroxide, while considering the effects of various metabolites using *Lactobacillus* strains that cannot synthesize lactate and hydrogen peroxide. This study has not been able to evaluate this issue. The effect of *Lactobacillus* supernatant on *C. albicans* biofilm formation and the change from yeast to hyphal form could be better understood by imaging evaluation using electron microscopy. However, due to equipment limitations, electron microscopic evaluation was not available for this study. In future studies, evaluation with images should also be considered.

In this study, quantitative evaluation of lactobacilli metabolite (lactate and hydrogen peroxide) concentrations revealed that the inhibitory effects of lactate and hydrogen peroxide on *C. albicans* might be acting through multiple stages, such as *C. albicans* growth, hyphal formation, biofilm development, and adhesion to epithelial cells. Therefore, combining antifungal drugs with lactobacilli as a live biotherapeutic product, with anti-biofilm development activity, may lead to the development of new treatment strategies. Future studies are required to evaluate how lactobacilli affect both *C. albicans* and NAC, to promote the global use of lactobacilli.

## Data availability statement

The original contributions presented in the study are included in the article/**Supplementary Material**. Further inquiries can be directed to the corresponding author.

## Author contributions

Conceptualization: TT and HaK. Methodology: HaK, KO, and TK. Software: SE. Validation: TT, AM, and HaK. Formal analysis: SE. Investigation: TT, SE, AM, and HaK. Resources: TT. Data curation: HaK. Writing-original draft preparation: TT, SE, AM, and HaK. Writing-review and editing: TT. Visualization: ES. Supervision: YukiY, MT, YukaY, HT, HM, and HiK. Project administration: TT. Funding acquisition: MT and HiK. All authors have read and agreed to the published version of the manuscript.
