# Supplementary material for: Inhibitory effects of vaginal Lactobacilli on Candida albicans growth, hyphal formation, biofilm development, and epithelial cell adhesion
Source: Front Cell Infect Microbiol. 2023 May 2;13:1113401. doi: 10.3389/fcimb.2023.1113401 (PMC10188118; doi:10.3389/fcimb.2023.1113401)
Supplement: Supplementary file 1 [file DataSheet_1.docx]

Supplementary Material

Inhibitory Effects of Vaginal Lactobacilli on *Candida albicans* Growth, Hyphal Formation, Biofilm Development, and Epithelial Cell Adhesion

Tomonori Takano, Hayami Kudo, Shuhei Eguchi, Asami Matsumoto, Kentaro Oka, Yukitaka Yamasaki, Motomichi Takahashi, Takuro Koshikawa, Hiromu Takemura, Yuka Yamagishi, Hiroshige Mikamo, Hiroyuki Kunishima*

*** Correspondence:** Hiroyuki Kunishima: [h2kuni@marianna-u.ac.jp](mailto:h2kuni@marianna-u.ac.jpe)

# Supplementary Figures

**Supplementary Figure 1.** The correlation between biofilm formation and growth speed of *C. albicans*. The biofilm formation of *C. albicans* HB-1, HB-10, LB-9 and LB-22 were used to measure WST-1 and growth speed of *C. albicans* were calculated by increased value of optical density at 600 nm and exhibited by correlation plot. Rho value (*r*) and *p* value calculated by Spearman’s rank correlation coefficient.

**Supplementary Figure 2.** The correlation between hydrogen peroxide concentration and lactate concentration, optical density at 600 nm value and lactate concentration and OD600 and hydrogen peroxide concentration of *Lactobacillus* sp. The correlation between lactate concentration and hydrogen peroxide concentration, lactate concentration and optical density at 600 nm and hydrogen peroxide concentration and optical density at 600 nm were exhibited by correlation plot. Rho value and p value calculated by Spearman’s rank correlation coefficient.

**Supplementary Figure 3.** The correlation between biofilm formation and lactate concentration and biofilm formation and hydrogen peroxide concentration of *Lactobacillus* sp. The biofilm formation of *C. albicans* HB-10 treated with *Lactobacillus* sp. culture supernatant were used to measure WST-1. The correlation between biofilm formation and lactate concentration and biofilm formation and hydrogen peroxide concentration were exhibited by correlation plot. Rho value and p value calculated by Spearman’s rank correlation coefficient.

**Supplementary Figure 4.** The biofilm formation of *C. albicans* HB-10 treated with lactate, hydrogen peroxide, or a combination of lactate and hydrogen peroxide. The biofilm formation of *C. albicans* HB-10 treated with lactate, hydrogen peroxide, or a combination of both, was used to measure WST-1 and was exhibited by the box whisker plots. The statistical analysis was performed with MRS group and the other group. *, *p* <0.05 by U-tests.

**Supplementary Figure 5.** The hyphal formation rate of C. albicans HB-10 treated with lactate and pH adjusted lactate. The hyphal formation rate is expressed relative to the MRS (control) and MRS pH adjusted (control). The statistical analysis was performed with the MRS group and normal group, MRS pH adjusted and pH adjusted group. The value exhibited on the bar chart was actual pH. *, *p* <0.05 by U-tests.

**Supplementary Figure 6.** The biofilm formation of *C. albicans* HB-10 treated with lactate and pH adjusted lactate. The biofilm formation of *C. albicans* HB-10 treated with lactate and pH adjusted lactate, was used to measure WST-1 and was exhibited by the box whisker plots. The statistical analysis was performed with MRS group and normal group, MRS pH adjusted and pH adjusted group. The value exhibited on the box whisker plot was actual pH. *, *p* <0.05 by U-tests.

**
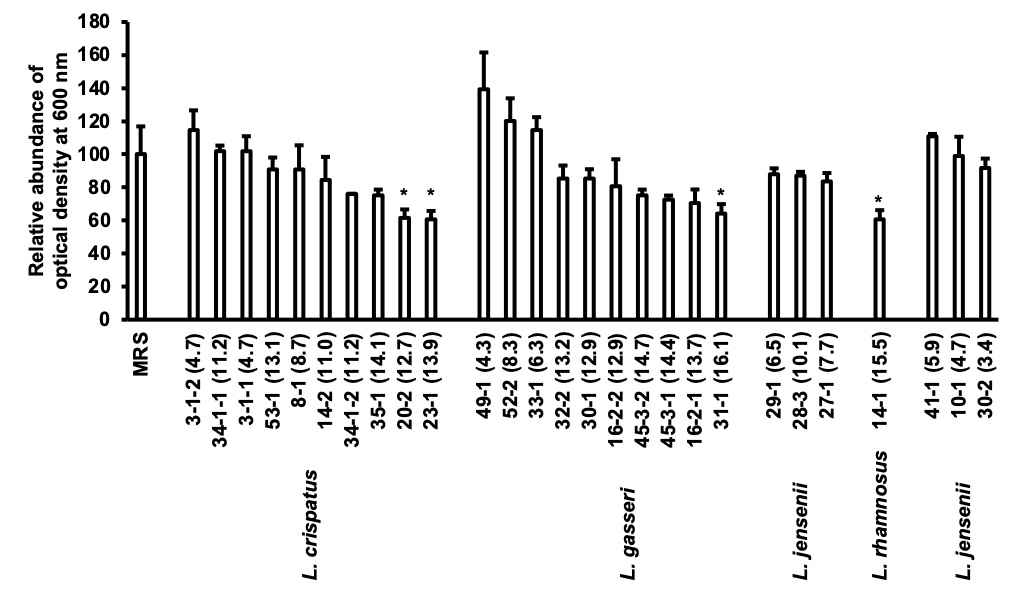
**

**Supplementary Figure 7.** The growth inhibition of *C. albicans* HB-10 treated with *Lactobacillus* culture supernatant. The optical density is expressed relative to the MRS (control) The statistical analysis was performed with MRS group and the other group. *, *p* <0.05 by U-tests.


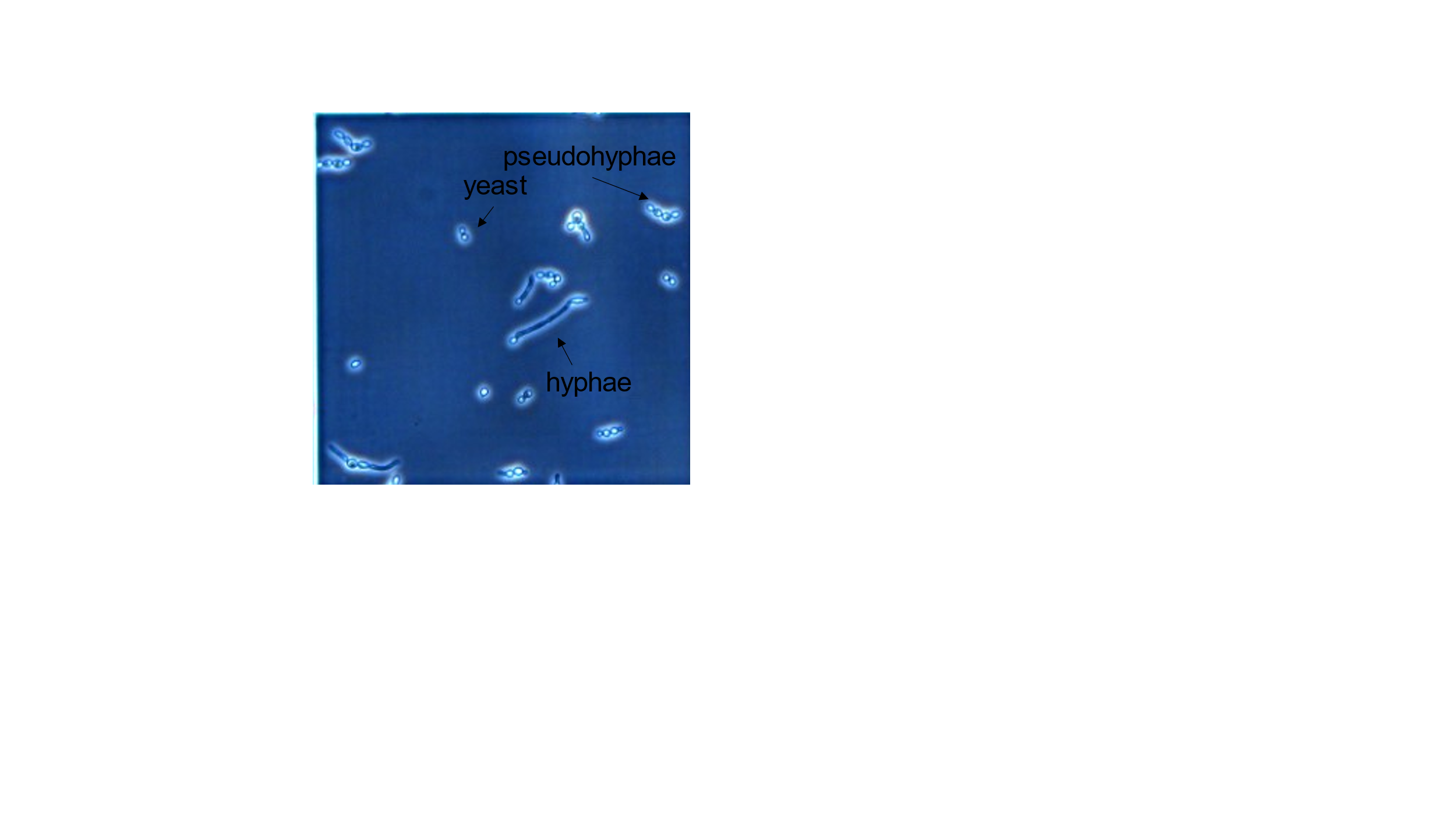


**Supplementary Figure 8.** The hyphal formation observed under light microscope. Under the light microscope, hyphae, pseudohyphal, and yeast forms were distinguished.

# Supplementary Tables

**Supplementary Table 1.** Characteristics of *Lactobacillus.*

| No. | Genus | Species | Strain denomination | Isolation date | Origin |
| --- | --- | --- | --- | --- | --- |
| 1 | *Lactobacillus* | *crispatus* | 3-1-1 | 2014/1/20 | Human vaginal discharge |
| 2 | *Lactobacillus* | *crispatus* | 3-1-2 | 2014/1/20 | Human vaginal discharge |
| 3 | *Lactobacillus* | *crispatus* | 8-1 | 2014/1/20 | Human vaginal discharge |
| 4 | *Limosilactobacillus* | *vaginalis* | 10-1 | 2014/1/20 | Human vaginal discharge |
| 5 | *Lacticaseibacillus* | *rhamnosus* | 14-1 | 2014/1/28 | Human vaginal discharge |
| 6 | *Lactobacillus* | *crispatus* | 14-2 | 2014/1/28 | Human vaginal discharge |
| 7 | *Lactobacillus* | *gasseri* | 16-2-1 | 2014/1/28 | Human vaginal discharge |
| 8 | *Lactobacillus* | *gasseri* | 16-2-2 | 2014/1/28 | Human vaginal discharge |
| 9 | *Lactobacillus* | *crispatus* | 20-2 | 2014/1/28 | Human vaginal discharge |
| 10 | *Lactobacillus* | *crispatus* | 23-1 | 2014/1/28 | Human vaginal discharge |
| 11 | *Lactobacillus* | *jensenii* | 27-1 | 2014/4/28 | Human vaginal discharge |
| 12 | *Lactobacillus* | *jensenii* | 28-3 | 2014/4/28 | Human vaginal discharge |
| 13 | *Lactobacillus* | *jensenii* | 29-1 | 2014/4/28 | Human vaginal discharge |
| 14 | *Lactobacillus* | *gasseri* | 30-1 | 2014/4/28 | Human vaginal discharge |
| 15 | *Limosilactobacillus* | *vaginalis* | 30-2 | 2014/4/28 | Human vaginal discharge |
| 16 | *Lactobacillus* | *gasseri* | 31-1 | 2014/4/28 | Human vaginal discharge |
| 17 | *Lactobacillus* | *gasseri* | 32-2 | 2014/7/28 | Human vaginal discharge |
| 18 | *Lactobacillus* | *gasseri* | 33-1 | 2014/7/28 | Human vaginal discharge |
| 19 | *Lactobacillus* | *crispatus* | 34-1-1 | 2014/7/28 | Human vaginal discharge |
| 20 | *Lactobacillus* | *crispatus* | 34-1-2 | 2014/7/28 | Human vaginal discharge |
| 21 | *Lactobacillus* | *crispatus* | 35-1 | 2014/7/28 | Human vaginal discharge |
| 22 | *Limosilactobacillus* | *vaginalis* | 41-1 | 2014/7/28 | Human vaginal discharge |
| 23 | *Lactobacillus* | *gasseri* | 45-3-1 | 2014/7/28 | Human vaginal discharge |
| 24 | *Lactobacillus* | *gasseri* | 45-3-2 | 2014/7/28 | Human vaginal discharge |
| 25 | *Lactobacillus* | *gasseri* | 49-1 | 2014/7/28 | Human vaginal discharge |
| 26 | *Lactobacillus* | *gasseri* | 52-2 | 2014/7/28 | Human vaginal discharge |
| 27 | *Lactobacillus* | *crispatus* | 53-1 | 2014/9/8 | Human vaginal discharge |

**Supplementary Table 2.** Primers used in this study.

| Target | Sequence (5'-3') | Tm (°C) | Length (bp) | Reference |
| --- | --- | --- | --- | --- |
| *ECE1* | Forward: CCAGAAATTGTTGCTCGTGTTGCCA | 62 | 140 | (Lee et al., 2021) |
|  | Reverse: TCCAGGACGCCATCAAAAACGTTAG | 61 |  |  |
| *HWP1* | Forward: TTGTTTGCGTCATCAAGACTTTG | 56 | 73 |  |
|  | Reverse : GTCTTCATCAGCAGTAACACAACCA | 59 |  |  |
| *YWP1* | Forward: GTTCCATTTTTCCAAGTTCATTTAG | 53 | 170 |  |
|  | Reverse : TCAAGAGTAGAACCTTCAAGAGCAG | 58 |  |  |
| *ACT1* | Forward: GTTGGTGATGAAGCCCAATC | 55 | 79 | (Gunsalus et al., 2016) |
|  | Reverse: CCCAGTTGGAAACAATACCG | 55 |  |  |

**Table S3.** Total cell counts and hyphal cell counts were listed in hyphal formation assay.

| species | strain No. or sample name | total cell count | hyphal cell count |
| --- | --- | --- | --- |
|  | MRS | 91 | 51 |
| *L. crispatus* | 35-1 | 104 | 19 |
|  | 53-1 | 130 | 28 |
|  | 3-1-1 | 79 | 36 |
|  | 14-2 | 115 | 55 |
|  | 3-1-2 | 97 | 53 |
| *L. gasseri* | 45-3-1 | 118 | 36 |
|  | 32-2 | 126 | 45 |
|  | 45-3-2 | 102 | 50 |
| *L. vaginalis* | 41-1 | 121 | 47 |
|  | 10-1 | 74 | 37 |
|  | Lactate 64 mM | 115 | 11 |
|  | Lactate 16 mM | 106 | 13 |
|  | Lactate 4 mM | 121 | 31 |
|  | H_2_O_2_ 64 nM | 104 | 37 |
|  | H_2_O_2_ 16 nM | 102 | 45 |
|  | H_2_O_2_ 4 nM | 109 | 52 |
